# Facilitation as an effective strategy to reduce excessive antibiotic prophylaxis in Children’s hospitals: A stepped-wedge cluster randomized controlled trial

**DOI:** 10.21203/rs.3.rs-6579066/v1

**Published:** 2025-05-30

**Authors:** Virginia McKay, Sara Malone, Emmanuel Tetteh, Jacqueline Saito, Shawn Rangel, Kelly Bono, Jade Tao, Jingxia Liu, Harry Obeng, Andrew Atkinson, Jason Newland

**Affiliations:** Washington University in St Louis George Warren Brown School of Social Work; Washington University School of Medicine in Saint Louis: Washington University in St Louis School of Medicine; Washington University in St Louis George Warren Brown School of Social Work; Children’s National Hospital; Boston Children’s Hospital Department of Surgery; Washington University in St Louis Division of Infectious Diseases; Washington University School of Medicine in Saint Louis: Washington University in St Louis School of Medicine; Washington University School of Medicine in Saint Louis: Washington University in St Louis School of Medicine; Washington University in St Louis George Warren Brown School of Social Work; Washington University School of Medicine in Saint Louis: Washington University in St Louis School of Medicine; Nationwide Children’s Hospital

**Keywords:** facilitation, de-implementation, antimicrobial stewardships

## Abstract

**Background:**

Excessive use of postoperative prophylactic antibiotics in children’s hospitals is a significant public health concern, leading to increased risks of infections like *Clostridioides difficile*, multidrug-resistant organisms, and unnecessary healthcare costs. Antibiotic stewardship programs (ASPs) are designed to optimize antibiotic use, but ideal strategies for implementing evidence-based guidelines remain unclear. We tested facilitation, a dynamic process where trained individuals support healthcare personnel in bridging evidence-practice gaps, as a promising strategy for the de-implementation of unnecessary postoperative antibiotics in healthcare.

**Methods:**

The OPerAtiC trial employed a stepped-wedge cluster randomized controlled design across nine hospitals to compare the effectiveness of two ASP-led strategies, specifically order set changes to align with antibiotic guidelines (baseline arm) and facilitation training (intervention arm). Facilitation workshops were informed by the i-PARIHS framework, emphasizing context analysis, evidence application, and recipient engagement; and were conducted remotely. Data were collected from 2019 to 2024, involving interviews with stewardship team members every two months. Data collected included proximal implementation outcomes of each ASP team member (acceptability, feasibility, appropriateness), intermediate outcomes (facilitation skill use) reported by the ASP team, and order set change completion rates.

**Results:**

Proximal implementation outcomes for both strategies were rated high across all study phases, indicating strong baseline enthusiasm among participants (N = 30). Key facilitation skills–effective communication, conflict resolution, and data presentation–were pivotal for successful implementation. Most order set changes (76%) were completed post-facilitation, targeting various specialties and achieving reductions or eliminations in antibiotic use. Facilitation was associated with significantly more completed order sets targeting antibiotic reduction (p = 0.01), thereby increasing the appropriateness of antibiotic use.

**Conclusions:**

Facilitation is a valuable approach in refining ASP efforts, contributing to the successful reduction and de-implementation of unnecessary antibiotic use in children’s hospitals. The study underscores the need for ongoing training and support for ASP teams to enhance their effectiveness in promoting appropriate antibiotic-prescribing practices. Future research should explore the long-term impacts of facilitation on antibiotic stewardship and patient outcomes

**Trial Registration information:**

ClinicalTrials.gov ID NCT04366440, https://clinicaltrials.gov/study/NCT04366440?term=OPERATIC&rank=1&tab=history, registered on 04/27/2020.

## Background

Over 200,000 surgical procedures are performed among children in the US annually, making it a common reason for hospitalization. Among hospitalized children, surgical patients receive 43% of all antibiotics delivered in children’s hospitals.^1^ Preoperative antibiotics allow surgeons to perform important, life-saving procedures while minimizing the likelihood of surgical site infections. However, postoperative antibiotics are often used prophylactically despite guidelines recommending against their administration for many surgeries.^2–5^ Studies, including our previous work, have shown that inappropriate postoperative antibiotic prophylaxis, specifically prolonged duration, occurs in up to 40% of cases, representing a substantial excess of antibiotic use in children’s hospitals.^6,7^

Excessive antibiotic use can have severe consequences for both patients and communities. Just a single dose of perioperative antibiotic prophylaxis increases risk of *Clostridiodes difficile* infection (CDI) six-fold, a life-threatening infection that can cause severe diarrhea and dehydration.^7,8,9^ Children who develop CDI while hospitalized are at increased risk of death, prolonged hospitalization by up to 7 days and increased hospital costs of approximately $100,000.^10^ Excessive antibiotic prescribing accelerates the emergence of multi-drug resistant (MDR) bacteria resulting in reduced antibiotic effectiveness, thereby generating larger public health concerns.^11–14^ By 2050, an estimated 39 million individuals worldwide will have died from an MDR bacterial infection.^40^

Antibiotic stewardship programs (ASPs) are a condition of participation for hospitals to receive funding by the Centers of Medicare and Medicaid Services to monitor and improve the use of antibiotics by helping to ensure high-quality and safe care for patients. These programs most frequently are co-led by physicians and pharmacists. Through clinical training, ASP stewards hold technical expertise regarding infectious diseases and the most appropriate avenues to prevent and treat infections using antibiotics. The Centers for Disease Control and Prevention (CDC) recommends that ASPs feature programs and strategies to optimize antibiotic use within hospitals, for example, through audits and feedback, education, and improving leadership abilities.^15^ Stewardship teams are expected to carry out these strategies by working with other clinical specialties and hospital staff like information technology (IT) staff to maximize optimal antibiotic use.

While strides have been made in developing ASPs, ideal strategies to *implement* evidence-based guidelines for antibiotic use are unclear, and some strategies lack a robust evidence base.^16^ For example, while the Infectious Diseases Society of America (IDSA) and Society for Healthcare Epidemiology of America antimicrobial stewardship guidelines recommend implementing interventions to reduce antibiotic therapy to the shortest effective duration,^17^ the best approaches to de-implement prolonged antibiotic courses are not well described. Furthermore, while such recommendations incorporate specific contextual factors related to the patient (e.g., severity of illness) and infection (e.g., bacterial species), these recommendations do not overtly consider other important contextual factors in which these strategies are implemented, such as the specialty service prescribing an antibiotic.^18^ Theoretical frameworks of ASP intervention delivery and our prior work both suggest that these contextual factors create variability in prescribing practices.^19^

Excessive antibiotic use aligns with the emerging field of de-implementation research within implementation science, which seeks to reduce the use of interventions (e.g., antibiotics) that are harmful or have no effect.^20,21^ This can be achieved by eliminating the intervention entirely, reducing the use of the intervention, using the intervention with a more limited target population, or replacing it with a different intervention.^22^ Within stewardship, all of these outcomes are of value since all lead to either the more appropriate use of effective antibiotics, like replacing more targeted antibiotics with more broad antibiotics (i.e., replacement), or overall reductions through total elimination (i.e., not using antibiotics at all), reduced antibiotic duration (i.e., using antibiotics for a shorter length of time), and limiting the cases for which antibiotics are used (i.e., targeted delivery).

A handful of strategies are known to be effective for de-implementation such as education, as well as many that are yet untested.^41,42^ Facilitation is emerging as a key strategy in healthcare implementation science to promote the successful uptake of evidence-based practices and interventions in clinical settings.^23,24^ Facilitation can be generally defined as a collection of strategies employed by an individual to support healthcare personnel in bridging the gap between evidence and practice.^25^ Facilitation is a dynamic process that involves the active engagement of the facilitating individual to work collaboratively with both individual providers and teams to identify and address barriers, foster relationships, and build capacity and momentum for change.^26^ The skills needed to serve in a facilitation role include organizational abilities and relationship-building in addition to clinical and technical expertise.^27,43^

Recent studies have demonstrated that individuals can be trained in facilitation skills and shed light on the potential value of facilitators in clinical settings.^27^ Facilitation in healthcare settings can range from external facilitators providing directive interventions in outpatient clinics to more intensive approaches where facilitators work closely with internal teams over many months to improve practices like post-operative pain management on surgical wards or nutritional care in places like nursing homes,^28,29^ suggesting facilitation as a potentially valuable strategy for addressing wide of implementation antimicrobial stewardship programs (ASPs). In addition, there are multiple parallels between ASPs and the functions of facilitators. ASPs encompass sets of directed interventions intended to optimize antimicrobial use without compromising patient health outcomes.^30^ Stewards work to implement evidence-based guidelines in the context of a complex interplay of factors, including provider behaviors, team dynamics, organizational culture, and system-level barriers.^31^

Improving prophylaxis in the postoperative period for children undergoing surgeries provides an excellent opportunity to reduce antibiotic overuse. Through a stepped-wedge, cluster randomized control trial, we tested two strategies employed by ASP teams, order set review with modification and facilitation training informed by implementation science to accomplish this goal. In this analysis, we report on the implementation outcomes from this trial to understand how ASP teams employed these strategies to effectively reduce antibiotic prophylaxis among low-risk surgeries in nine children’s hospitals.

## Methods

**Figure 1:**
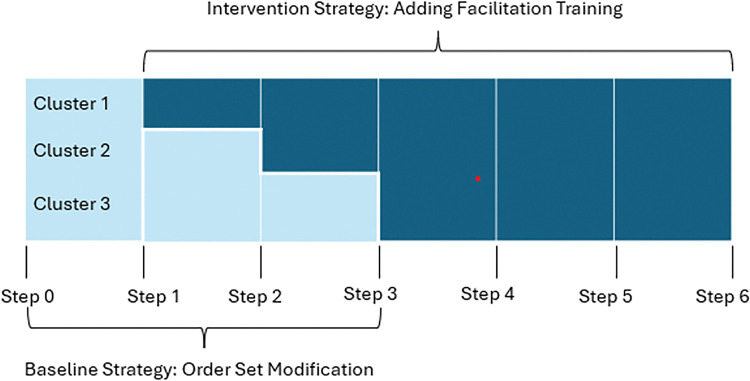
OPERATIC Study Design.

The Optimizing PERioperative AnTibiotICs ((OPerAtiC) trial (clinicaltrials.gov registration # NCT04366440) is a stepped-wedge, cluster randomized control trial with nine hospitals that are members of the SHaring Antimicrobial Reports for Pediatric Stewardship (SHARPS) Collaborative and the American College of Surgeons National Surgical Quality Improvement Project-Pediatrics (NSQIP-P).^32,33^ Hospitals were randomized into three clusters (three hospitals per cluster).^34^ This trial consisted of six steps lasting six months with all clusters starting in the baseline intervention arm on November 1, 2020 (See Fig. 1). The first cluster was switched to the intervention arm of the trial and participated in the facilitation workshop at step 2, the second cluster at step 3, and the third cluster at step 4. Data were collected bi-monthly, focusing on both implementation outcomes and clinical outcomes. For the current analysis, we report on multiple implementation outcomes measured throughout the trial. More details about the overall trial design and data collection approach can be found in our previously published protocol.^35^ The following methods are reported using CONSORT and STARI guidelines (provided as a Supplement). Data are available from the first author upon reasonable request.

### Recruitment and eligibility.

All hospitals in this study have an active ASP team that includes, at a minimum, a pediatric infectious diseases physician and pharmacist. Hospitals provided a letter of support prior to the start of the study indicating interest in participation and received annual compensation for participating in the study. ASP team members from the nine hospitals were eligible to participate in this study. Site leads at each hospital were identified and were responsible for providing a roster of ASP team members at their site. The study team then emailed each of the team members and invited them to participate in the facilitation training. These individuals included physicians, pharmacists, nurses, and other individuals affiliated with the ASP teams.

### Conceptual Model and Strategies.

#### Baseline intervention.

The baseline intervention was an educational session conducted remotely where the study team provided a description of the study design and data collection, a review of the evidence-based guidelines for surgical prophylaxis, and a request to change order sets to align with guidelines conducted with all ASP teams in November 2020. We did not specifically target specialties, procedures, or antibiotics.

#### Enhanced intervention.

**Figure 2:**
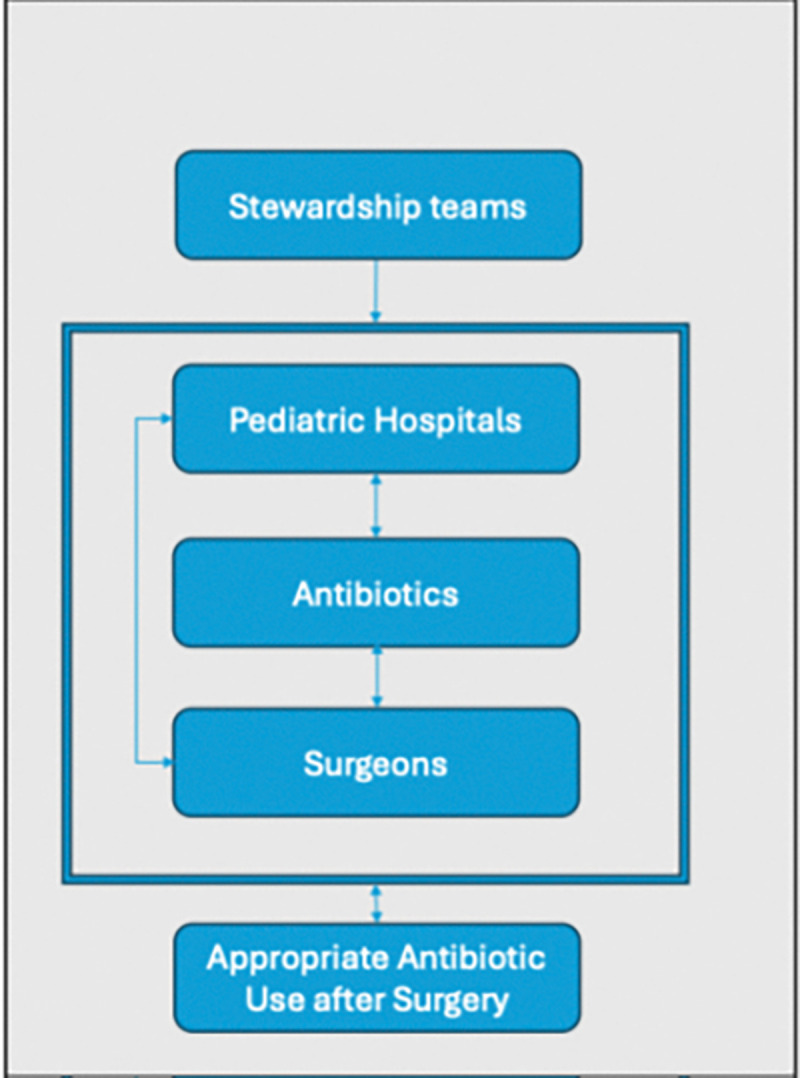
Conceptual model based on i-PARHIS framework.^46^

As hospitals were moved into the intervention arm of the trial, ASP teams participated in a facilitation workshop. The workshop was conducted remotely using Zoom^48^ and consisted of four, two-hour sessions conducted weekly in May 2021 (Step 1), November 2021 (Step 2), and May 2022 (Step 3). The overall goal of the sessions was to train ASP team members to act as facilitators within their setting based on the i-PARHIS framework (Fig. 2).^37^ This framework posits that facilitators impact three major domains to enhance evidence-based practice. First, the contextual setting where evidence-based interventions are embedded, the nature of the relevant evidence, and the recipients who will ultimately use or be impacted by the evidence-based intervention. In the context of antimicrobial stewardship to optimize surgical prophylaxis, we considered the context to be multi-level including surgical departments and the hospital at large. The relevant evidence, in addition to evidence-based guidelines, was hospital-specific infection epidemiology and prophylaxis utilization as well as perceptions of antibiotic usage among surgeons based on our prior work.^39^ Finally, surgeons and their teams were the primary individuals to be targeted by ASP teams, although we recognized that others may influence decision-making and standards around antibiotic use. Sessions focused specifically on fostering better working relationships with surgeons and presenting evidence in a compelling manner to different individuals who influence decisions around antibiotic usage, including surgeons, department heads, and other hospital administrators. The content was both didactic and activity-based. More specific details about the training, including content details, participant evaluation, and aspects used from the training over time are described elsewhere.^38^

#### Data Collection.

Data were collected from 2020–2024. Data were collected via two methods; first, we conducted a baseline survey of all hospitals and then ongoing surveys of ASP team members. Surveys were completed via REDCap survey, and a link was emailed to each team member at each data collection point. The survey asked about implementation outcomes that are indicative of being able to achieve the implementation outcome. These data were collected for both order set changes from baseline and for facilitation when hospitals were included in the intervention arm.

Second, a member of the research team conducted interviews with the leads of the ASP teams at each site. These interviews consisted of closed and open-ended questions. One member of the stewardship team was interviewed every two months from baseline to year four of the project. The interview consisted of two broad sections: 1) Use of facilitation skills, which we conceive as an intermediate implementation outcome. These data were collected exclusively from hospitals once in the intervention arm. 2) Order set changes that include changes in surgical prophylaxis usage on the order set, which constitutes the most distal implementation outcome. These data were collected from baseline throughout the data collection period.

#### Measures.

The baseline survey of each hospital’s ASP program evaluated the characteristics of the programs (e.g., dedicated clinician time, existing programmatic efforts, and expertise of team members). Ongoing surveys of ASP team members included our proximal implementation outcome consisted of three measures of both the strategies, order set changes and facilitation: the Acceptability of Intervention Measure (AIM), the Feasibility of Intervention Measure (FIM), and the Intervention Appropriateness Measure (IAM), which we will collectively refer to as AIM/FIM/IAM.^36^ This collection of measures was focused specifically on evaluating order set changes and facilitation as strategies to implement evidence-based guidelines. All items were on a Likert-type scale from 1–5 with 5 indicating a high degree of that element (i.e., a high level of capability). To measure use of facilitation skills, our intermediate implementation outcome, participants were asked if they used specific facilitation skills covered in the workshop. Additionally, individuals were asked at the final data collection point to reflect on which skills they felt were most valuable from the facilitation training through an open-ended question. Finally, participants were asked about their efforts to change order sets including whether the order set was new or revised, specialty targeted, surgery targeted, date order set completed, antibiotic targeted, duration change, or any other change associated with the order set.

### Analysis

Data were collected and managed in REDCap and analyzed in R (4.4.1). The data were analyzed in phases. The data were cleaned and examined for missingness. Descriptive statistics were generated and are presented for the baseline hospital and ASP programs.

We analyzed proximal implementation outcomes across all time points. The time points were aligned with respect to placement in control or intervention as opposed to the date of data collection, such that pre-intervention and post-intervention data were analyzed separately. Missing data was assumed to occur at random in analyses. Univariate. Descriptive statistics were calculated, with frequency and mean (SD) or median (IQR) calculated as appropriate for each measure and examined across each time point. Additionally, these data were analyzed with respect to each institution. Open-ended questions related to facilitation skills were analyzed descriptively.

#### Order set changes.

Order sets were initially analyzed descriptively. After descriptive statistics were examined, the data was analyzed with respect to the significant number of order set changes pre and post-intervention. We ran a pre-post analysis on order set changes that were completed solely before the intervention or after, removing all changes completed that occurred in between the two phases. This was performed due to our qualitative data suggesting that the virtual facilitation aided the completion of the order set changes. We used the non-parametric Wilcoxon signed rank test with continuity correction due to the small sample size.

## Results

Table 1 provides the characteristics of hospitals. In terms of ASP teams, all hospitals had dedicated time for stewardship physicians and pharmacists (N = 9), and most had dedicated time from a data analyst (N = 8). The median number of beds in the hospitals at baseline was 372 (250–859), and the most common electronic medical record systems were Epic (67%) and Cerner (33%). Pre-existing prophylactic guidelines differed by specialty, with general surgery having the highest prevalence (88%), followed by orthopedic, neurosurgery, and urology (all 56%). Before the trial, prophylactic data were shared with surgeons by two-thirds (67%) of hospitals, usually once a year or twice a year. Only 33% of the ASP teams have ever seen an NSQIP-P report, despite just over half (56%) of hospitals being aware of NSQIP-P at the start of the study.

### Proximal Implementation Outcomes – Acceptability, Appropriateness, and Feasibility of Strategies.

We evaluated the acceptability, appropriateness, and feasibility of order set changes and facilitation. Results for each of these including the mean and median are included in Table 2. Briefly, for order set changes, participants rated all three relatively high at all three points in the study phase. We observed a statistically significant change in acceptability with a .5 change in the median score from the baseline to the final step (baseline 4.5 to 5.0 at the final step, p = 0.03). Similarly, for facilitation, participants rated the strategy high at both the first intervention step and the final step, but no significant change was observed. Appropriateness significantly increased by .25 from the first intervention step and the final step (p = 0.01).

### Intermediate Outcomes – Facilitation Skills

During final interviews, staff suggested three major facilitation skills that promoted the overall success of implementation order sets in their hospitals. The first, effective communication and collaboration with surgical teams, was observed by staff as paramount for successful facilitation, with one clinician emphasizing that “success depended on strong relationships with surgical specialties.” The second and closely related to the first, learning about different communication styles and team conflict resolution strategies, was also recognized as valuable. Another clinician reflected positively upon workshops that “normalized” different conflict techniques and drew awareness to different communication styles. Lastly, several staff drew attention to using the presenting data thoughtfully. One provider found that “sharing data [back to surgeons about prophylaxis use] was most helpful,” while another clinician highlighted the importance of “educating people on guidelines and data” and “using local data to drive change.” These results suggest that different types of evidence were valuable depending on the circumstance.

### Order Set Outcomes

Approximately 76% of order set changes attempted were completed (66/87). Hospitals attempted on average 9 order set changes (range 0–19) and completed an average of 7 order set changes (range 0–17). Table 3 describes completed order set changes, demonstrating a variety of specialties targeted and a focus on reducing prophylaxis duration. Only 8 order set changes were initiated and completed in the pre-intervention period. Thirty-three were started and completed post-intervention, while the remaining 25 were initiated pre-intervention and completed post-intervention. In addition, 76% of the order sets were revised with 42 of them reducing antibiotics and 34 eliminating antibiotics for post-operative prophylaxis. When examining the likelihood of completing order sets in alignment with evidence-based guidelines, teams were significantly more likely to successfully complete order sets after facilitation training, even after excluding order sets initiated prior to facilitation training but completed afterward (p = .01).

**Figure 3:**
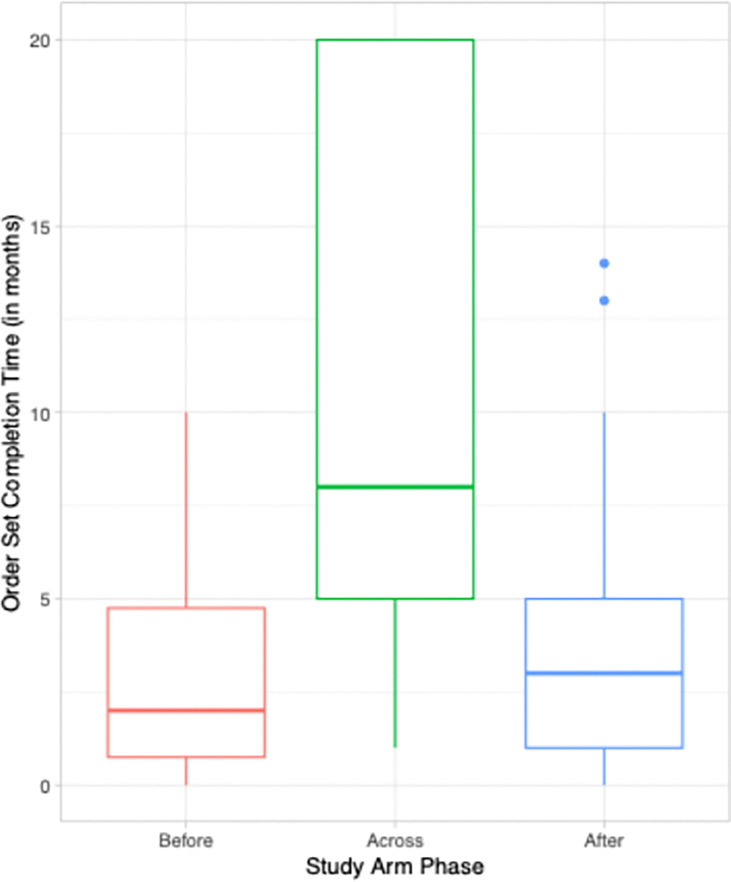
Time taken to complete order set changes in months.

To further elucidate the amount of effort involved in completing an order set, Fig. 3 shows a boxplot of the time taken to complete order set changes once initiated in months by study arm. Overall, the median time to complete an order set change was 5 months (range 1–20 months). Because some order sets were initiated prior to moving into the enhanced intervention (i.e., facilitation) arm of the trial, we separated order sets into three groups: those initiated and completed before facilitation training, “Before”, those initiated before, but completed after facilitation training, “Across”, and those initiated and completed after facilitation training “After”. Order set changes entirely before or entirely after facilitation training were similar in their time from initiation to completion (a median of 2 months [1–4.75 months], and a median of 3 months [1–5 months], respectively. Order set changes that spanned from pre- to post-facilitation training took much longer with a mean of 8 months.

### Other relevant outcomes.

Individuals also reported other activities to support change beyond order set changes. This included having informal agreements with clinicians, building dashboards to understand antibiotic usage in the hospital better, and doing educational interventions to help increase knowledge about surgical prophylaxis guidelines among clinical staff.

## Discussion

Excessive antibiotic use is of growing public health concern, and children’s hospitals are a critical setting in which excessive use occurs. Simultaneously, evidence supporting de-implementation strategies in any health area is limited, with many strategies yet to be examined. Given the nexus of ASPs, in the context of children’s hospitals, we sought to test facilitation training as a strategy to reduce unnecessary postoperative surgical prophylaxis.

We did not observe a change in proximal implementation outcomes. There may be several explanations for this finding. First, participants, ASP team members, were already highly enthusiastic about working towards reduced antibiotic use as they were members of the SHARPS Collaborative, a group dedicated to improving antibiotic use in children.^44^ In other words, they were already highly motivated and felt they had ample opportunity and capability to do the work. Furthermore, the overall approach of both order sets and facilitation were perceived by the ASP teams as good strategies to address prophylaxis. An alternative explanation is that the measures were not sufficiently sensitive to the variability that existed in these concepts.

We demonstrated that ASP teams were able to bring surgical order sets into alignment with current evidence about antibiotic prohpylaxis more effectively after facilitation training than when using order set changes as a strategy alone. Most notably, the wide majority of order sets were completed post-facilitation training, suggesting that an appropriate strategy like changing order sets is not sufficient alone to motivate change. Facilitation’s specific mechanisms of action and effectiveness in promoting the success of medical interventions remain understudied, especially in the context of complex department- or organization-wide interventions.^2,3^ When examining facilitation skills, participants reported consistent use over the course of the study, and these results are reported elsewhere.^38^ However, we were able to explore which skills were the most critical to support successful order set changes from the perspective of our participants. Participants indicated that many of the “soft skills” associated with successful facilitators were the skills that they found most helpful, such as communication skills and data presentation skills. These findings align with our preliminary work highlighting the necessity of good relationships and communication between ASP teams and surgeons as a facilitator to implementing new guidelines to reduce excessive antibiotic use.^39^ We suggest that these may be important mechanisms within this context which served to build rapport and relationships between those working to implement (i.e., ASP teams) and those primarily impacted by the change (i.e., the surgeons). Taken together with the lack of changes in proximal outcomes and in alignment with our guiding theoretical model,^46,47^ these results suggest that while participants were interested in creating change, doing so is not simple in the context of children’s hospitals where excessive antibiotic use is the result of clinician behavior, local epidemiological evidence impacting antibiotic effectiveness, and overall contextual factors, like departmental and hospital leadership, which can constraint clinical practice. The premise that facilitation is a beneficial strategy is further supported by the observation that other unanticipated activities that are known to foster a positive context for implementing evidence-based clinical care, like dashboards, which support the monitoring of relevant outcomes were developed and potentially act as mechanisms of action.^25^

### Achieving de-implementation.

Our results indicate that facilitation as a complement to other strategies is effective for de-implementation given a complex set of barriers. We observed a wide range of specialties, procedures, and antibiotics targeted. We also observed a range of de-implementation outcomes including elimination, reduction, and replacement (i.e., antibiotic switch). We did not give specific targets at the start of the study, rather, we encouraged participants to accomplish what they felt was feasible based on their local context, given the difficulty with successfully accomplishing de-implementation observed in other studies,^41^ and our prior work that suggested surgeons perhaps needed time to emotionally adjust and that incremental change was positive. We were pleased with these outcomes since all brought the intended practice into closer alignment with evidence-based guidelines. From a public health perspective, overall reductions and more appropriate antibiotic use are desirable, regardless of whether they are completely eliminated or reduced, as a means to ensure that they continue to be effective long-term. However, for other interventions, it may be more critical to have specific de-implementation outcomes depending on the intended health impact.

### Implications for stewardship.

ASPs are situated to optimize antibiotic use, and clinicians who comprise these programs have extensive technical expertise and recommended strategies to support their goals towards optimizing antibiotic use. However, bringing practice into alignment with the evidence base, in the case of reducing excessive antibiotic use within pediatric hospitals, requires fostering behavior change among other clinicians. A recent study demonstrated that high-performing ASPs in adult hospitals had strong relationships between physicians and the ASP team stressing the importance of interactions that our facilitation skills training aided.^45^ Our trial provides support for facilitation and specific facilitation skills as an approach to successfully reducing antibiotic use in children’s hospitals through enhancing the delivery of other effective ASP strategies including prospective-audit-and-feedback, clinical practice guidelines, and diagnostic stewardship interventions. Supporting the efforts of ASP teams is critical given the myriads of potential targets and strategies that programs might implement.

### Limitations

There are several notable limitations to the study, which should be taken into consideration. First, all measures are self-reported. Participants may have not recalled all instances of applying facilitation skills for instance. However, we would expect that our most critical implementation outcome, order sets, are less likely subject to recall bias because of the frequency of the data collection and the difficulty with which this strategy was accomplished. Second, our study reflects a relatively small number of hospitals and ASP teams, making it difficult for us to conduct more robust analyses.

## Conclusions

Facilitation training of the ASP team as a method to enhance order set changes is an effective approach to reduce excess antibiotic use in children’s hospitals. Facilitation aided the ASP team in many ways including aiding them in developing better relationships and collaborations through the understanding of communication and conflict styles. Hospitals are complex settings with numerous individuals important to providing the best, evidence-based care. The development of different strategies, especially those that aid in strengthening relationships like facilitation, will be important for both implementation and de-implementation.

## Figures and Tables

**Table 1:** Hospital Characteristics at Baseline (N = 9)

Hospital Characteristics	
Number of Beds Median (Range)	372 (250–859)
Electronic Medical Record, n (%)	
Epic	6 (67%)
Cerner	3 (33%)
ASP FTE Support, n (%)	
Physician	9 (100%)
Pharmacist	9 (100%)
Analyst	9 (100%)
Nurse Practitioner	9 (100%)
Previously Existing Prophylaxis Guidelines, n (%)	
Otolaryngology	4 (44%)
General surgery	8 (88%)
Neurosurgery	5 (56%)
Plastic Surgery	4 (44%)
Orthopedic Surgery	5 (56%)
Urology	5 (56%)
Do you share prophylaxis data with surgeons? n (%)	
Yes	6 (67%)
No	3 (33%)
If yes, how often? n (%)	
Quarterly	1 (17%)
Twice a year	2 (33%)
Once a year	2 (33%)
Other	1 (17%)
Are you aware of NSQIP-P? n (%)	
Yes	5 (56%)
No	4 (44%)
Have you ever seen a NSQIP-P report? n (%)	
Yes	3 (33%)
No	6 (67)

**Table 2:** Proximal implementation outcomes for strategies to de-implement excessive post-surgical prophylaxis (1 = completely disagree, 5= completely agree) p-value pertains to the change from baseline to final step from the Wilcoxon signed rank test (with continuity correction).

	Baseline	1st intervention step	Final step	P value
Order set changes as a strategy				
Acceptability	4.46 (4.5)	4.71 (4.88)	4.69 (5.00)	**0.03**
Appropriateness	3.88 (3.75)	3.82 (3.75)	4.01 (4.00)	0.09
Feasibility	4.5 (4.5)	4.71 (5)	4.65 (5.00)	0.57
Facilitation as a strategy				
Acceptability	**-**	4.42 (4.63)	4.44 (4.5)	0.43
Appropriateness	**-**	3.78 (3.75)	4.06 (4.00)	0.01
Feasibility	**-**	4.47 (5.00)	4.45 (4.5	0.30
Mean, (Median)					

**Table 3:** Description of Completed Order Set Changes (N = 66)

	n	%
Surgical Specialty Targeted	
Neurosurgery	20	30.3
General	14	21.1
Plastic	11	16.7
Cardiology	9	13.6
Urology	5	7.6
Orthopedics	4	6.1
Other	3	4.5
Type of Revision	
New	16	24.0
Revised	50	76.0
Prophylaxis Change Made*	
Elimination	34	44.0
Reduction	42	88.0
Antibiotic Switch	9	9.0
Change timing	
Pre-facilitation only	8	12.0
Pre-to-Post facilitation	25	38.0
Post-facilitation only	33	50.0

* Order sets may have made multiple changes and will not add to 100%.

## Data Availability

Data are available from the lead author upon request

## Abbreviations

MDROs: Multi-drug-resistant organisms
ASPs: Antibiotic stewardship programs
CDC: Centers for Disease Control and Prevention
IT: Information technology
IDSA: Infectious Diseases Society of America
OPerAtiC: Optimizing PERioperative AnTibiotICs
SHARPS: SHaring Antimicrobial Reports for Pediatric Stewardship
NSQIP: P-National Surgical Quality Improvement Project-Pediatrics
i-PARIHS: Integrated-Promoting Action on Research Implementation in Health Services
AIM: Acceptability of Intervention Measure
FIM: Feasibility of Intervention Measure
IAM: Intervention Appropriateness Measure
CONSORT: Consolidated Standards of Reporting Trials
STARI: Standards for Reporting Implementation Studies
